# Editorial: Differentiation and Regulation of Bone Marrow Mesenchymal Stromal Cells

**DOI:** 10.3389/fmolb.2022.950930

**Published:** 2022-07-13

**Authors:** Hao Zhang, Lipeng Wang, Xiao Chen

**Affiliations:** ^1^ Department of Orthopedics, Shanghai Changhai Hospital, Naval Medical University, Shanghai, China; ^2^ Institute of Translational Medicine, Shanghai University, Shanghai, China; ^3^ Musculoskeletal Organoid Research Center, Shanghai University, Shanghai, China

**Keywords:** bone marrow mesenchymal stromal cells, differentiation, regulation, bone disease, osteoporosis

Bone marrow mesenchymal stromal cells (BMSCs, also known as bone marrow mesenchymal stem cells) are a group of heterogeneous stromal cells with various differentiation potentials contributing to the postnatal genesis of osteoblasts, chondrocytes, and adipocytes as well as the maintenance of hemopoietic niches. Under pathological conditions like osteoporosis, osteoarthritis, etc., the differentiation shift of BMSCs has gained attention and been ever in the spotlight in recent decades. This Research Topic focuses on the mechanisms and regulations of BMSCs differentiation as well as therapeutic strategies for altering BMSC differentiation shifts for bone diseases. This Research Topic contains 16 reports, 5 literature reviews, and 11 pieces of original researche.

Identification and isolation of BMSCs is the first step to study the differentiation of BMSCs. Gao et al. comprehensively reviewed the current knowledge about several new subgroups of BMSCs. They could be divided into multipotent stem cells and skeletal stem cells, and the former cells are expected to mainly differentiate into adipocytes while the latter are the stem cells of osteoblast and chondrocyte. The differential lineages of skeletal stem cells were also compared between humans and mice in this review. Wei et al. identified and isolated a new subtype of MSCs derived from lateral mesoderm (LM-MSCs) using a novel protocol. LM-MSCs are similar to BMSCs but showed a homeodomain transcription factor (HOX) gene expression pattern. This new type of MSCs showed increased osteogenic and chondrogenic differentiation capacity and hematopoietic support potential compared to BMSCs. LM-MSCs might be an ideal cell source for bone and blood diseases after further confirmation.

Insight into mechanisms controlling BMSCs differentiation is also interesting. Differentiation of BMSCs in normal organisms may be related to epigenetic and gene expression. The contribution by Liu et al. provided updated insights into the role of epigenetic modifier proteins, chromodomain helicase DNA-binding (CHD) proteins, in BMSCs. CHD proteins promote disruption of histone-DNA contacts and exhibit regulatory effects in stem cell proliferation, differentiation, and functioning. It is involved in neurodevelopmental disorders, including CHARGE syndrome and Kallmann syndrome, cancers, and bone diseases. Thus, epigenetic regulation in stem cells could be a clinical target.

The development of high-throughput sequencing technologies and transcriptomics has provided new methods to create a deeper understanding of key genes in BMSC differentiation. Data from rat BMSC differentiation high-throughput transcription factors sequencing presented by Zhang et al. demonstrated that Hopx and other early responder TFs may control the osteogenic cell fate of BMSCs and participate in the development of osteoporosis. Gbx2 and other early responder TFs should be considered in mechanistic models that clarify the anabolic changes in cartilage during the clinical progression of osteoarthritis. As was shown by Liu et al., Ccna2, Cdc20, and Il6 may act as common hub genes in initiating osteogenesis, adipogenesis, and chondrogenesis. Expression of Mex3b, Sertad1, and Hopx was only increased throughout three early phases during the osteogenic differentiation. Dtx4 and Ibsp expression occurred in adipogenesis and chondrogenesis, respectively. Bioinformatics makes it easier to screen key genes in BMSC differentiation. In human, Sun et al. found a key gene in BMSC osteogenic differentiation *via* bioinformatics-guided analysis. Aldehyde oxidase (AOX) was screened out in his study which showed a strong association with osteoblast-related pathways. AOX increased during BMSCs differentiation and AOX overexpression significantly increased the expression of osteo-specific genes. More transcriptome sequences of BMSCs in different species should be down to piece together a panorama of gene regulation in BMSC differentiation.

BMSCs reside in a complex microenvironment with multiple cells including adipocytes, immunocytes and osteoclasts. Cross-talks between BMSCs and different neighbors regulate BMSCs differentiation and exert important physiological action. In the review contributed by Gao et al., cross-talks occurred between bone and fat to control the bone mass through several pathways. RANKL and adipokines secreted by adipo-lineage cells negatively regulate BMSC osteogenic differentiation. The role of fat in the bone marrow and its regulatory effect on BMSCs was highlighted in the review article by Wang et al. Bone marrow adipocytes (BMAs) are critical regulators in hematopoiesis, osteogenesis, and osteoclastogenesis. As a cell type derived from BMSCs, BMAs inhibit osteoclast formation *via* bone morphogenetic protein receptor (BMPR) and epidermal growth factor receptor pathways and adipokines. An inflammatory response is essential for bone formation and BMSC osteogenic differentiation during fracture healing. Deng et al. uncovered that macrophages could express four kinds of osteoinductive cytokines, including BMP2, FGF2, TGFβ3, and OSM, under co-culture with hucMSC-derived extracellular matrix (hucMSC-ECM). They believed that macrophage-mediated osteogenesis depended on MIF/CD74 signal transduction. Crosstalk between osteoclast and osteoblast has been well investigated, and the OPG-RANK-RANKL axis is essential in coupling bone remodeling. Though a prior study (Xie et al., 2014) revealed that pre-osteoclast could increase angiogenesis *via* PDGF-BB to regulate BMSCs osteogenic differentiation, direct crosstalks between pro-osteoclast and BMSCs remain mysterious. Data presented by Bai et al. demonstrated tartrate acid phosphatase (TRAP)-positive monocytes secreted CTGF to activate BMSCs in periosteum during bone regeneration. Usually, pre-osteoclasts are TRAP-positive monocytes. CTGF derived from pre-osteoclast promoted bone healing by activating BMSCs and directing lineage commitment directly. Cross-talks between BMSCs and related cells could provide novel clinical targets in curing fracture and osteoporosis.

Differentiation of BMSCs is precisely regulated by a variety of factors including genes, microenvironment, and metabolism. In pathological conditions, differentiation of BMSCs is disturbed, leading to a decrease in tissue repair capacity and worsening disease. Diabetes mellitus (DM) is widely known for its susceptibility to multiple complications in the elderly. Xu and Zuo reviewed the research of stem cell fate alteration under DM in depth. Hyperglycemia of DM patients induced both dysfunctions and quantity alteration of stem cells *via* diabetic microenvironment. Stem cells showed abnormal mobilopathy, differentiation, migration, and secretion because of a rising level of oxidative stress and pro-inflammatory factors. Lipid metabolism also influences BMSCs differentiation. As was discovered by Lin et al., lipid availability could modulate the osteogenesis of skeletal progenitors. They found that lipoprotein receptor-related protein 5 (LRP5) participated in lipid uptake during BMSC osteogenesis. Lipid scarcity or Lrp5 ablation could decrease bone quality and suppresses BMSC osteogenic differentiation. Normal glucose and lipid metabolism are essential for maintaining stem cell proliferation and differentiation, but the changes in stem cell differentiation in different systemic diseases still need to be further explored.

Osteogenic differentiation ability of BMSCs plays a vital role in the development of orthopedic diseases. Exploring the mechanisms of osteogenic differentiation of BMSCs and developing therapeutic strategies are the current hot topics of research on BMSCs. In this Research Topic, Ni et al. investigated that IL-18 promoted the osteogenic differentiation of hBMSCs *via* the SLC7A5/c-MYC pathway. This research provided a possible pathway of IL-18 in promoting bone formation, but they only provided *in vitro* data. The contribution by Xu et al. provided both *in vitro* and *in vivo* evidence that Zinc finger E-box-binding homeobox 1 (ZEB1) could regulate osteogenesis. Silencing of ZEB1 in BMSCs promoted osteogenic activity and mineralization partly through the Wnt/β-catenin pathway, which is a major pathway regulating BMSC osteogenic differentiation. In-depth mechanistic studies provide therapeutic targets for regulating the differentiation of BMSCs, but target-specific drug development remains challenging. Small molecules derived from traditional Chinese medicine could regulate the differentiation of BMSCs. Wang et al. investigated that curculigoside (CCG), a bioactive component of Curculigo orchioides, could regulate the bone-fat balance in the marrow of aging mice *via* transcriptional co-activator with PDZ-binding motif (TAZ) expression.

Overall, the studies included in this Research Topic expand our knowledge of BMSCs subtype. The differentiation process and several key regulatory genes of BMSCs in physiological status have been revealed in three studies. The shift of BMSC differentiation in some pathological conditions were explored and reviewed by articles in this Research Topic. Osteogenic differentiation of BMSCs as a main clinical translation point was well researched in this topic, and several key regulatory proteins and novel drugs were developed to cure related bone diseases.
